# Supplementary material for: Identifying the multiple drivers of cactus diversification
Source: Nat Commun. 2024 Aug 23;15:7282. doi: 10.1038/s41467-024-51666-2 (PMC11343764; doi:10.1038/s41467-024-51666-2)
Supplement: Supplementary file 1 — Supplementary Information [file 41467_2024_51666_MOESM1_ESM.pdf]

Supplementary information for "Identifying the multiple drivers of cactus diversification"

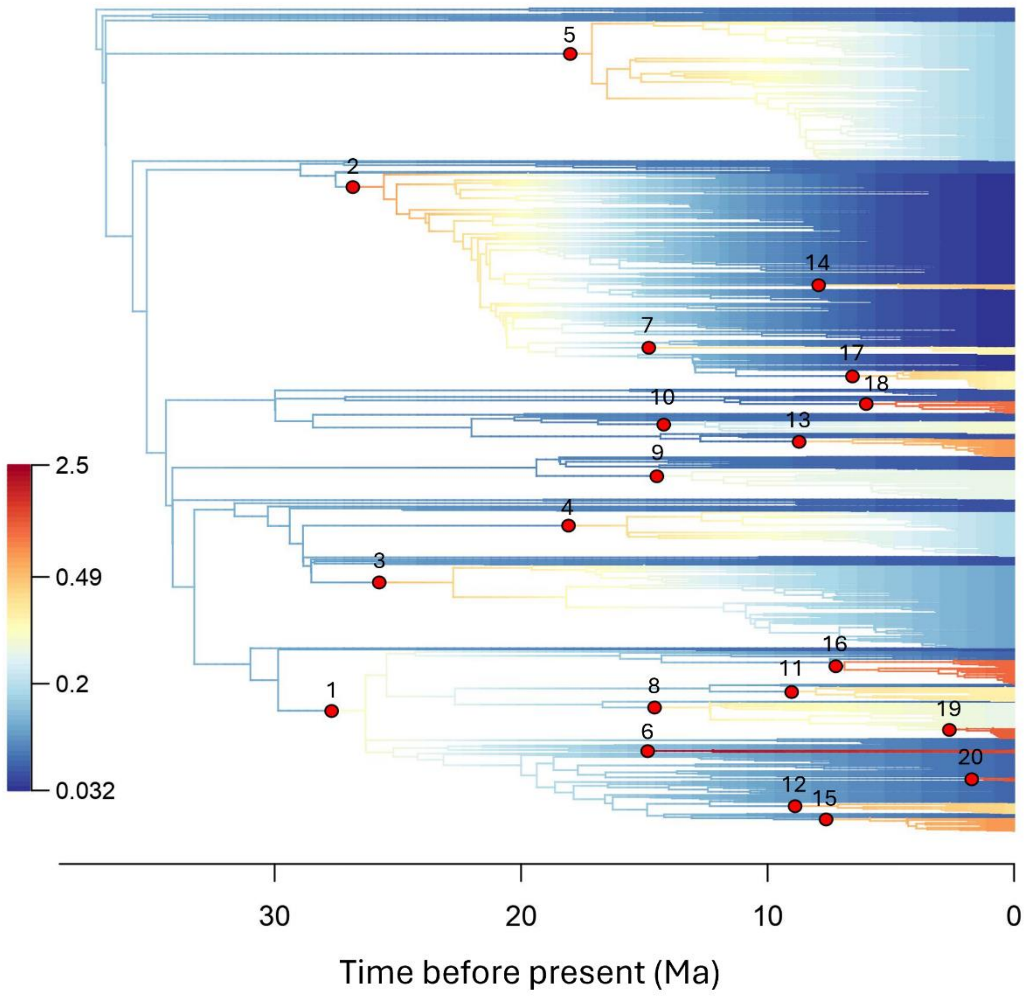

Supplementary Figure 1: The maximum a posteriori rate shift configuration estimated by BAMM, with branches coloured by speciation rate, and rate shifts (red circles). Numbers above nodes are associated with rate shift information in Supplementary Table 1.

Supplementary Table 1: Temporal, sampling, and taxonomic details of lineages experiencing rate shifts. All are accelerations, and node number corresponds to rate shifts in Supplementary Figure 1.

**Node number**	**Time (Mya)**	**Number of sampled genera and species**	**Description**
1	27.68	38, 233	Cereeae
2	26.83	21, 279	Cacteae (excluding *Aztekium*, *Geohintonia*, *Echinocactus*, *Astrophytum*)
3	25.76	11, 107	Echinocereeae
4	18.08	7, 68	Hylocereeae
5	18.00	16, 180	Opuntioideae
6	14.87	1, 5	*Rebutia*
7	14.83	1, 10	*Mammillaria*
8	14.58	8, 47	*Cereus, Pilosocereus, Brachycereus, Jasminocereus, Cipocereus, Coleocephalocereus, Espostoopsis*
9	14.49	1, 38	*Rhipsalis*
10	14.22	1, 16	*Parodia*
11	9.01	5, 19	*Cereus, Pilosocereus, Brachycereus, Jasminocereus, Cipocereus*
12	8.89	1, 14	*Echinopsis*
13	8.17	1, 23	*Eriosyce*
14	7.92	1, 7	*Coryphantha*
15	7.62	1, 18	*Echinpsis*
16	7.24	1, 32	*Gymnocalycium*
17	6.56	2, 24	*Mammillaria* ands *Coryphantha*
18	6.00	1, 16	*Copiapoa*
19	2.63	1, 14	*Pilosocereus*
20	1.71	1, 5	*Harrisia*

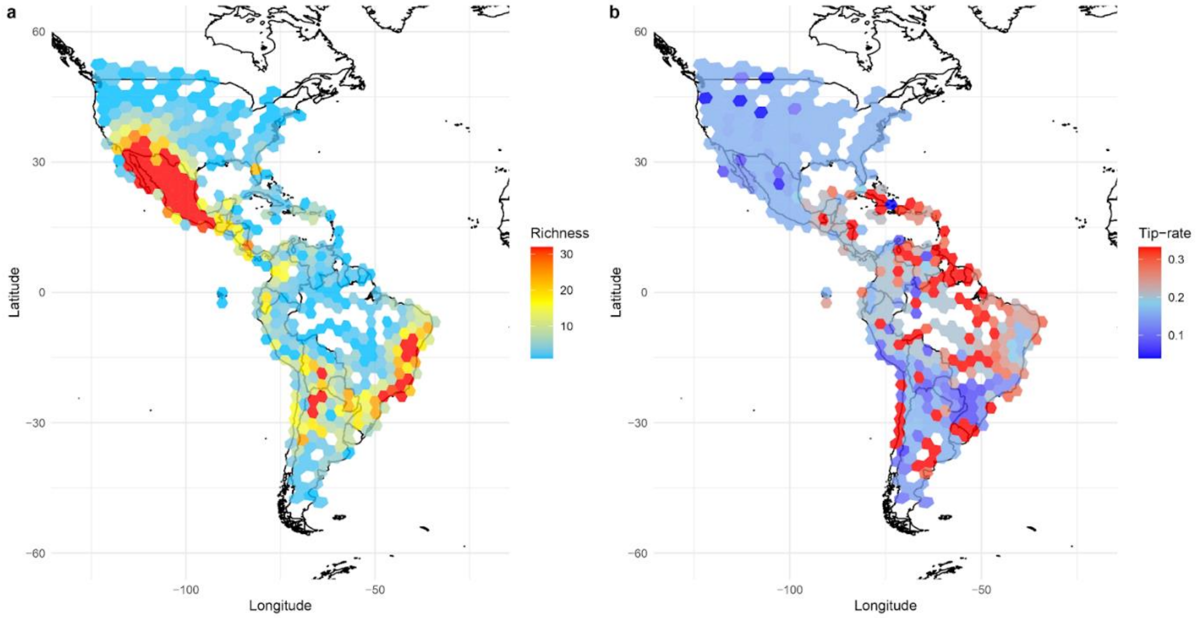

Supplementary Figure 2: Spatial variation in species richness and speciation rates of Cacti across the Americas. Species richness (a) and median tip speciation rate (b) are estimated for equal-area grid cells (~322km^2). Areas of highest richness (e.g. Mexico, Northern Argentina) do not necessarily have the fastest median speciation rates. Areas of low richness can have rapid speciation rates, such as in the Caribbean. This decoupling is documented throughout the tree of life, including on global scales in angiosperms (Igea and Tanentzap, 2020) and marine fish (Rabosky et al., 2018), and local island scales in plants (Messerschmid et al., 2023), including in island analogues (montane regions, Hughes and Eastwood, 2006).

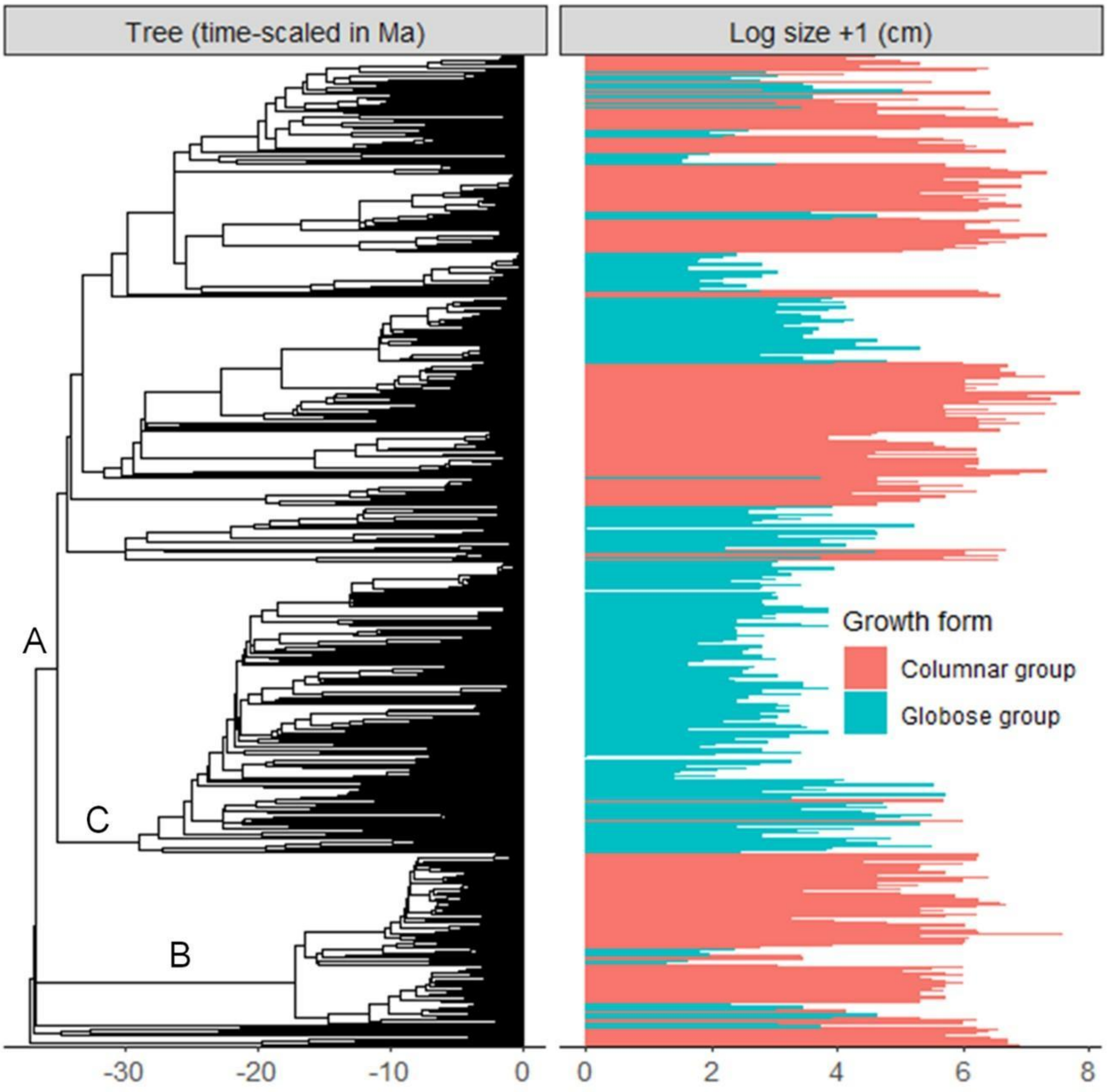

Supplementary Figure 3: The phylogenetic distribution of cactus size (plant height or length, in log cm+1), and growth form variation. Letters denote major lineages: A is subfamily Cactoideae, B is subfamily Opuntioideae, and C is tribe Cacteae (containing the important Mammilloid complex).

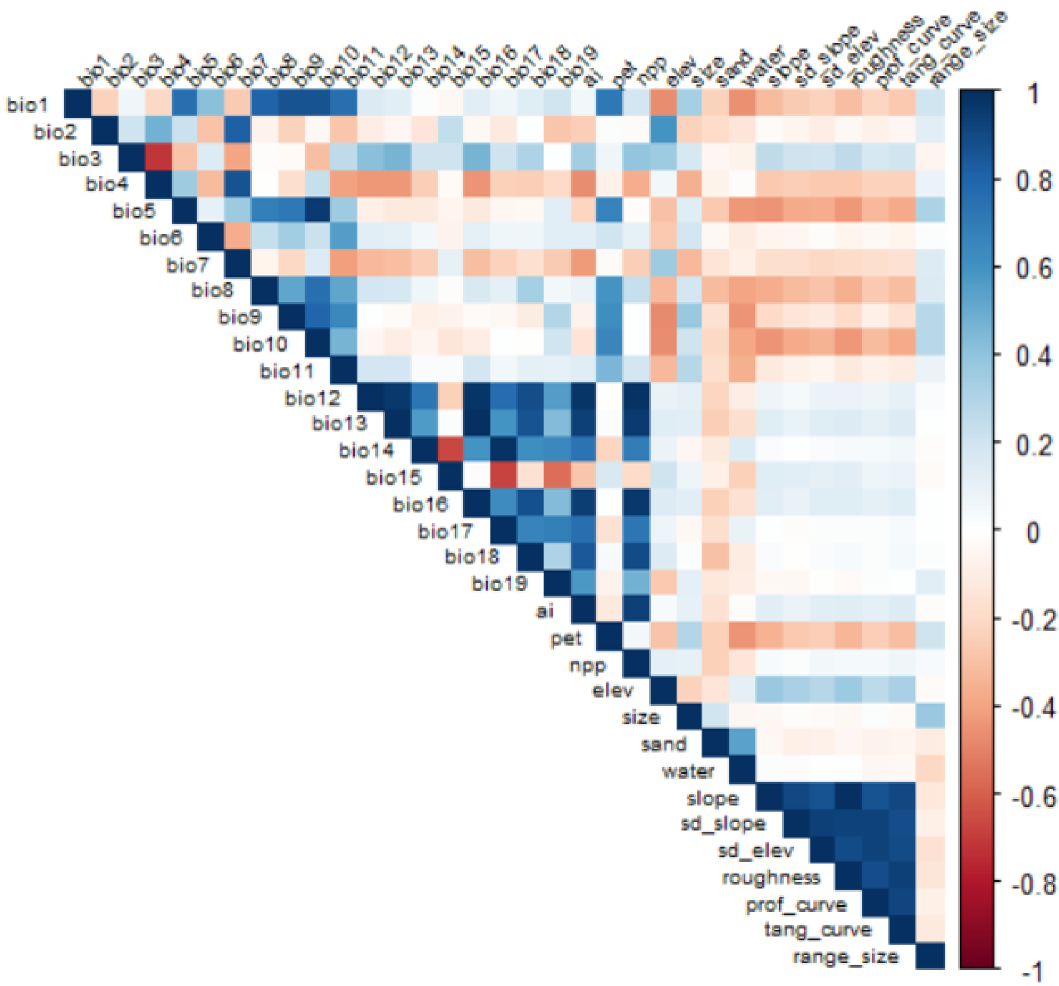

Supplementary Figure 4: Correlations between all continuous variables included in the XGBoost models show some evidence of strongly positive (r >0.7) and negative (r <-0.7) correlations.

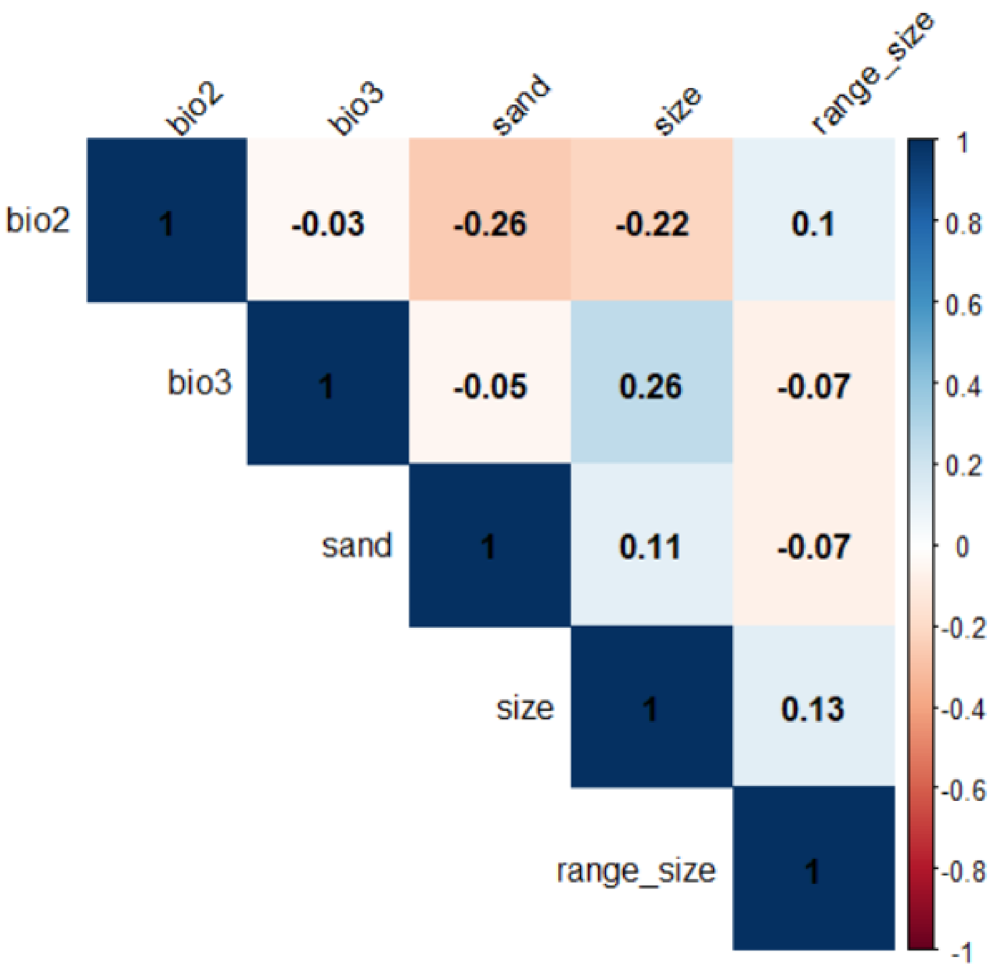

Supplementary Figure 5: Correlations between variables estimated as significant predictors of cactus speciation rate by XGBoost show no evidence of strong correlations (r >0.7 or <-0.7).

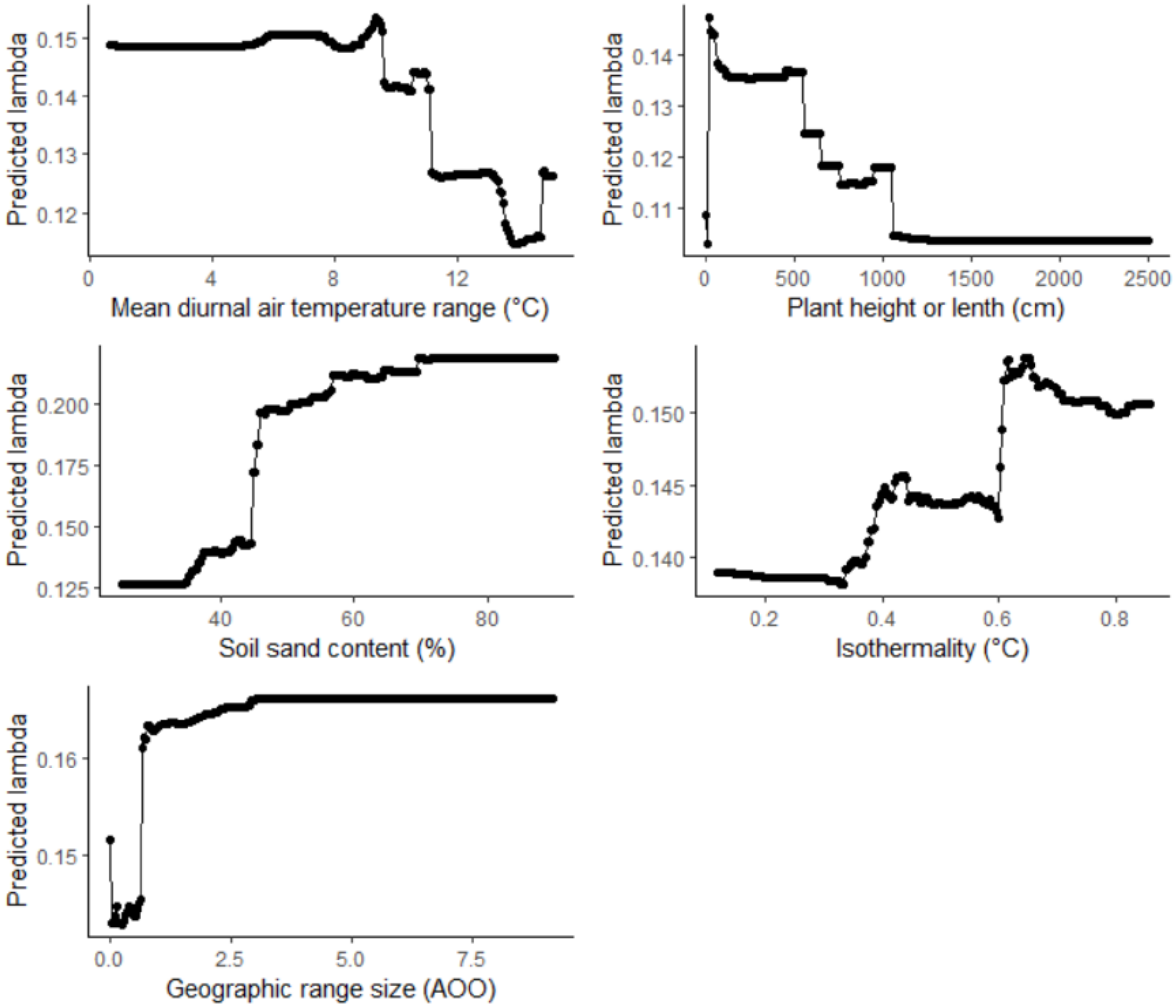

Supplementary Figure 6: Predicted speciation rate (lambda) across observed variation of the variables identified as significant in the full XGBoost model. In these predictive analyses, other variables are kept as median (if continuous) and mode (if discrete).

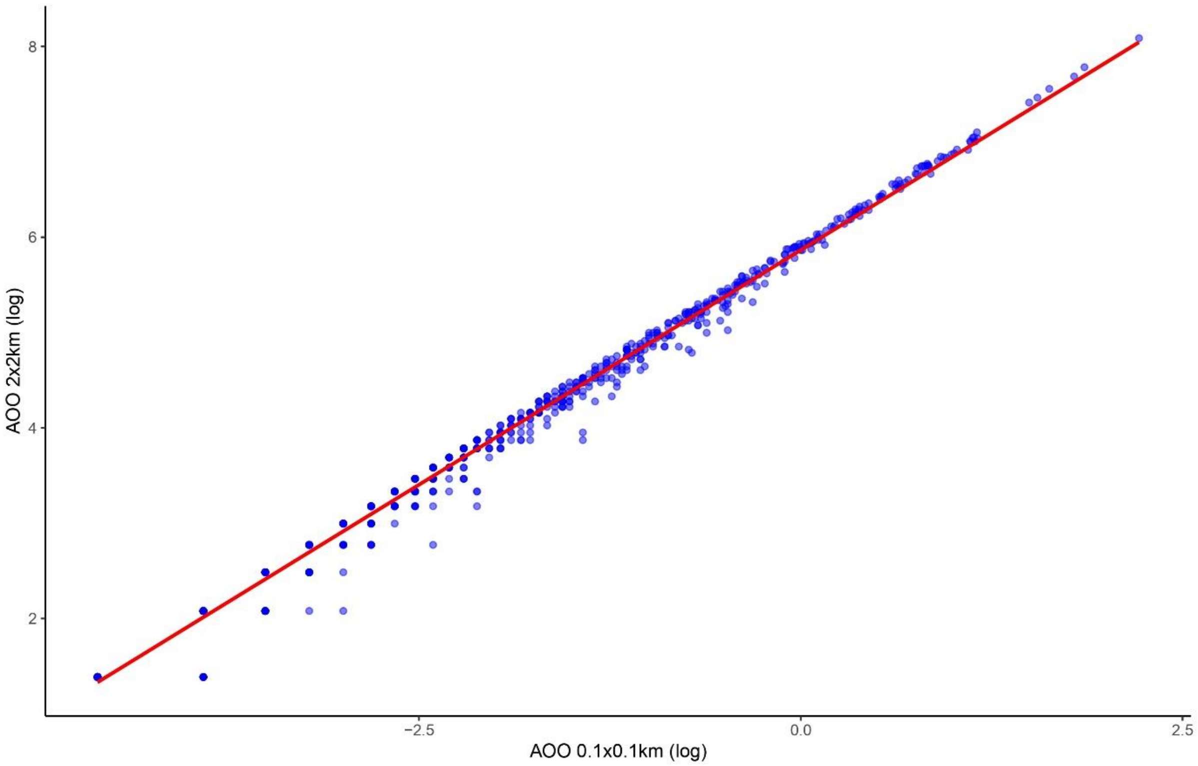

Supplementary Figure 7: A near perfect correlation between geographic range size (AOO) estimated with two grid cell sizes. The 0.1x0.1km cell size used in the analyses, to account for the small range sizes observed in cacti, and the 2x2km grid cell size recommended by IUCN.

Supplementary Table 2: Variables included in the XGBoost model, and how they may impact diversification rates.

Variable	Hypothesis
**Biotic**	
Chromosome count	Variations in chromosome count can lead to genetic incompatibilities and reproductive isolation, and whole-genome duplications can facilitate divergence among species.
Growth form	Creates distinct ecological strategies and interactions with pollinators and other species, potentially leading to reproductive isolation as each growth form adapts to specific environmental pressures and opportunities.
Plant size	Speciation processes are influenced by plant size (and its correlated variables), including seed dispersal, generation time and population size.
Pollination syndrome	Reproductive isolation increases due to the coevolution of floral traits and pollinator preferences, that reinforce fidelity to certain pollinators and reduce gene flow with other populations.
Epiphytism	The unique ecological niche of growing on other plants exposes epiphytic cacti to distinct microclimates and biological interactions, potentially leading to reproductive isolation through specialized adaptations to these aerial habitats.
**Abiotic (climatic)**	
Aridity index	Variations in aridity index could lead to divergent selection pressures, driving reproductive isolation as cactus populations adapt to different levels of water availability e.g. through differing levels of succulence.
Biome	Populations adapt to the specific biotic and abiotic factors unique to their respective biomes, from deserts to tropical forests.
Bio1 (annual mean temperature):	Warmer environments may accelerate diversification rate, due to higher evolutionary rates (mutations, metabolism, generation times).
Bio2 (mean diurnal range)	Populations that adapt to wider temperature fluctuations within a day may develop unique morphological and physiological traits, leading to reproductive isolation from populations in areas with less diurnal variation.
Bio3 (isothermality)	Populations adapted to environments with a consistent thermal niche (high isothermality) may evolve distinct physiological and temporal traits that facilitate reproductive isolation from populations in environments characterized by more pronounced temperature variability (low isothermality), where different survival strategies are selected.

Bio4 (temperature seasonality)	Reproductive isolation may occur among populations that are temporally and physiologically adapted to distinct seasonal climates.
Bio5 (maximum temperature of warmest month)	Species that evolve tolerance to peak seasonal temperatures may become reproductively isolated, due to the development of unique heat stress response mechanisms and associated life-history traits adapted to the upper thermal limits.
Bio6 (minimum temperature of coldest month)	Could lead to adaptations to the lowest temperatures experienced annually, potentially leading to reproductive isolation as populations diverge in cold tolerance mechanisms and thermal niche preferences.
Bio7 (temperature annual range)	Species adapted to areas with a broad temperature range between the coldest and warmest times of the year may develop distinct morphological and physiological traits, fostering reproductive isolation from those in regions with narrower temperature fluctuations.
Bio8 (mean temperature of wettest quarter)	Species may adapt mechanisms for water uptake and storage during periods of peak precipitation combined with temperature conditions, which may lead to reproductive isolation as populations become specialized to these specific climatic conditions.
Bio9 (mean temperature of driest quarter)	Thermal conditions during the driest part of the year can drive selection for drought survival strategies, potentially leading to reproductive isolation among populations that evolve distinct adaptations to these dual stress periods.
Bio10 (warmest quarter mean temperature)	Extreme temperatures during the warmest part of the year can select for distinct thermal tolerance thresholds and survival strategies, potentially fostering genetic divergence among populations adapted to different heat stress conditions.
Bio11 (coldest quarter mean temperature)	Populations become genetically adapted to the thermal conditions of their local winter climates, leading to divergence in physiological and survival traits.
Bio12 (annual precipitation)	Availability of water resources is critical for cactus survival and reproduction. This can lead to reproductive isolation as populations adapt to distinct water regimes and can lead to adaptive divergence and speciation.
Bio13 (precipitation of wettest month)	Species may evolve specialized succulent adaptations to capture and utilise episodic and abundant rainfall, leading to reproductive isolation among populations that are differentially adapted to the intensity and timing of peak precipitation events.
Bio14 (precipitation of driest month)	Scarce water availability could lead to the development of distinct drought-adaptation strategies, potentially resulting

	in reproductive isolation as these populations become more specialized to their particular arid conditions.
Bio15 (precipitation seasonality)	Variable seasonal rainfall patterns might select for different water-use strategies and flowering times across populations, leading to reproductive isolation through temporal mismatches in reproductive cycles and the evolution of divergent ecological niches.
Bio16 (precipitation of wettest quarter)	Species may evolve specialized succulent adaptations to capture and utilise episodic and abundant rainfall, leading to reproductive isolation among populations that are differentially adapted to the intensity and timing of peak precipitation events.
Bio17 (precipitation of driest quarter)	Scarce water availability could lead to the development of distinct drought-adaptation strategies, potentially resulting in reproductive isolation as these populations become more specialized to their particular arid conditions.
Bio18 (precipitation of warmest quarter)	Populations adapt to seasonal water availability during periods of high temperature, leading to reproductive isolation as populations diverge in their physiological responses to the dual stresses of heat and potential drought.
Bio19 (precipitation of coldest quarter)	Populations evolve distinct adaptations to manage water in cooler climates, potentially affecting flowering times and pollinator interactions.
Net primary productivity	Populations in areas with higher net primary productivity may have more resources to support a variety of life-history strategies, potentially leading to reproductive isolation through ecological specialization.
Potential evapotranspiration	Reproductive isolation increases as species adapt to different levels of water stress and develop specialized physiological traits to conserve or utilise water efficiently, in their respective microclimates.
**Abiotic (topographic/edaphic)**	
Elevation	Different altitudes present unique environmental conditions and selective pressures, leading to reproductive isolation as populations adapt to varying levels of ultraviolet radiation, temperature, and oxygen availability associated with altitude.
Profile curvature	Populations adapt to the resultant microhabitat variations in water availability caused by varying water runoff and soil moisture retention, which is critical for survival and reproduction in arid environments, resulting in reproductive isolation.
Roughness	Creates a mosaic of microhabitats with varying exposure to sun, wind, and water, leading to reproductive isolation as populations become adapted to the specific challenges

	and opportunities presented by their immediate rugged terrain.
Slope	Influences soil depth, moisture, and exposure to environmental elements, leading to reproductive isolation as populations adapt to the unique physical demands and ecological niches presented by their specific sloped environments.
Soil sand content	Variations in sand content affect soil drainage and aeration, leading to reproductive isolation as populations develop specialized root systems and physiological adaptations for different water and nutrient acquisition strategies in sandy versus less sandy soils.
Soil texture	Affects root penetration, water retention, and nutrient availability, potentially leading to reproductive isolation as cacti species adapt their growth forms and reproductive strategies to the specific soil texture of their habitats.
Soil water content	Reproductive isolation increases as populations evolve distinct water-use efficiencies and root morphologies to thrive in their respective soil moisture regimes.
Standard deviation of elevation	Creates a range of microclimatic conditions within a geographic area, leading to reproductive isolation as populations adapt to the diverse temperature, moisture, and solar radiation conditions.
Standard deviation of slope	Varying slope conditions lead to distinct microhabitats with different soil stability and water drainage patterns, potentially leading to reproductive isolation due to the adaptation of cacti to these specific terrain conditions.
Tang curvature	Affecting local water flow and erosion processes, potentially leading to reproductive isolation as cacti populations adapt to the micro-topographical variations that shape their immediate environmental conditions.
**Abiotic (miscellaneous)**	
Geographic range size	Larger geographic range sizes increase reproductive isolation via population fragmentation, as the opportunity to encounter barriers and new habitats increases.

References:

Hughes, C. and Eastwood, R. (2006). Island radiation on a continental scale: exceptional rates of plant diversification after uplift of the Andes. Proceedings of the National Academy of Sciences, 103(27), pp.10334-10339.

Igea, J. and Tanentzap, A.J. (2020). Angiosperm speciation cools down in the tropics. Ecology letters, 23(4), pp.692-700.

Messerschmid, T.F., Abrahamczyk, S., Bañares-Baudet, Á., Brilhante, M.A., Eggli, U., Hühn, P., Kadereit, J.W., Dos Santos, P., De Vos, J.M. and Kadereit, G. (2023). Inter-and intra-island speciation and their morphological and ecological correlates in *Aeonium* (Crassulaceae), a species-rich Macaronesian radiation. Annals of Botany, 131(4), pp.697-721.

Rabosky, D.L., Chang, J., Title, P.O., Cowman, P.F., Sallan, L., Friedman, M., Kaschner, K., Garilao, C., Near, T.J., Coll, M. and Alfaro, M.E. (201). An inverse latitudinal gradient in speciation rate for marine fishes. Nature, 559(7714), pp.392-395.
